# Tailoring the Size of Reduced Graphene Oxide Sheets to Fabricate Silicon Composite Anodes for Lithium-Ion Batteries

**DOI:** 10.1021/acsami.4c03710

**Published:** 2024-05-22

**Authors:** Yun-Zhen Liang, Ting-Yu Hsu, Yu-Sheng Su

**Affiliations:** † Industry Academia Innovation School, National Yang Ming Chiao Tung University, 1001 Daxue Road, Hsinchu 300093, Taiwan; ‡ International College of Semiconductor Technology, National Yang Ming Chiao Tung University, 1001 Daxue Road, Hsinchu 300093, Taiwan

**Keywords:** graphene oxide, spray drying, graphene-coated silicon, Li-ion channels, size-dependent, energy storage

## Abstract

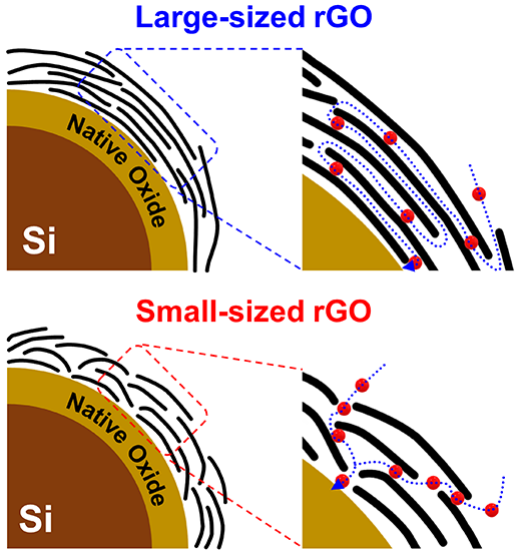

The integration of a silicon (Si) anode into lithium-ion batteries (LIBs) holds great promise for energy storage, but challenges arise from unstable electrochemical reactions and volume changes during cycling. This study investigates the influence of reduced graphene oxide (rGO) size on the performance of rGO-protected Si composite (Si@rGO) anodes. Two sizes of graphene oxide (GO(L) and GO(S)) are used to synthesize Si@rGO composites with a core–shell structure by spray drying and thermal reduction. Electrochemical evaluations show the advantages of the Si@rGO(S) anode with improved cycle life and cycling efficiency over Si@rGO(L) and pure Si. The Si@rGO(S) anode facilitates the formation of a stable LiF-rich solid electrolyte interface (SEI) after cycling, ensuring enhanced capacity retention and swelling control. Rate capability tests also demonstrate the superior high-power performance of Si@rGO(S) with low and stable resistances in Si@rGO(S) over extended cycles. This study provides critical insights into the tailoring of graphene-protected Si composites, highlighting the critical role of rGO size in shaping structural and electrochemical properties. The Si material wrapped by graphene with an optimal lateral size of graphene emerges as a promising candidate for high-performance LIB anodes, thereby advancing electrochemical energy storage technologies.

## 1 Introduction

The use of silicon (Si) as an anode material for lithium-ion batteries (LIBs) has great potential due to its high theoretical capacity (4,200 mA h g^–1^), making it an attractive candidate for next-generation energy storage systems.^1,2^ However, the practical implementation of Si anodes is hindered by inherent challenges, primarily due to the significant volume expansion and contraction that occurs during repeated lithiation and delithiation cycles.^1,3^ This mechanical stress results in the pulverization of Si particles, the formation of an unstable solid electrolyte interface (SEI), and consequently limited cycle life. To address these challenges, the incorporation of a protective layer, such as reduced graphene oxide (rGO), has emerged as a viable solution due to the low cost, highly dispersible, and facile reduction of graphene oxide (GO).^4−10^ The unique properties of rGO, including its mechanical strength, electrical conductivity, and flexibility, make it an ideal candidate for mitigating the adverse effects of Si volume changes, thereby improving the overall performance and lifetime of Si-based anodes.^5,11^

The motivation of this study is to meticulously explore the role of rGO in protecting Si anodes and, more importantly, to investigate how the size variations of rGO films affect the electrochemical performance of the LIBs. By systematically examining the pros and cons of Si as an anode material, elucidating the protective mechanisms conferred by the rGO layer, and delving into the influence of rGO size on battery characteristics, this research seeks to unravel the intricacies of reduced graphene oxide/silicon composite (Si@rGO) anodes. The tailored design of Si composites, in which the size of the rGO layers is systematically varied, provides a unique opportunity to fine-tune the protective properties of the rGO layer. Ultimately, this research is expected to provide important insights into the optimization of graphene-based composite electrode materials, paving the way for the development of high-performance and long-cycle-life LIBs for various applications.

## 2 Experimental Section

### 2.1 Preparation of Reduced Graphene Oxide/Silicon Composite Materials

To synthesize the Si@rGO composites, two different sizes of GO (The Sixth Element) in dispersion were utilized, referred to as GO(L) for large-sized GO and GO(S) for small-sized GO. The fabrication process consisted of mechanically stirring and ultrasonically dispersing 2 g of GO dispersion (solid content), 8 g of silicon powder, 0.05 g of single-wall carbon nanotubes (SWCNTs; OCSiAl TUBALL), and 0.1 g of poly(vinyl alcohol) (PVA; SHOWA) in deionized water. The resulting slurry was processed in a spray dryer where a controlled outlet temperature of 110 °C converted the slurry droplets into solid composite particles. The spray-dried powder was then subjected to GO reduction in a tube furnace at 950 °C in argon for 1 h at a ramp rate of 10 °C min^–1^.

### 2.2 Materials Characterizations

Scanning electron microscopy (SEM; Hitachi SU-8010) and transmission electron microscopy (TEM; JEOL JEM-2100F) were used for microstructural analysis. Energy dispersive X-ray spectroscopy (EDX) integrated with TEM facilitated elemental mapping and provided detailed insights into elemental distributions and material morphology. Particle size distribution was determined via dynamic light scattering (DLS) using an Otsuka ELSZ-2000 instrument with anhydrous ethanol dispersion. Thermogravimetric analysis (TGA; TA Instruments) included a heating ramp up to 800 °C in air. For crystallographic studies, X-ray diffraction (XRD; Bruker D2 Phaser) used Cu Kα radiation over a range of 10° to 60°. Raman spectroscopy (Princeton SP2750) using a high-resolution 532 nm laser provided GO/rGO analysis. The surface area and pore size distribution of the composites were determined using nitrogen adsorption gas by Brunauer–Emmett–Teller and Barrett–Joyner–Halenda methods (BET/BJH; Micromeritics ASAP 2020). X-ray photoelectron spectroscopy (XPS; ULVAC-PHI Quantera II) was used to perform a depth profile analysis of the composite anodes with an equipped argon ion gun (1 kV) to determine their composition at different locations.

### 2.3 Electrode Fabrication and Cell Assembly

The anode formulation consisted of 70% active material, 10% conductive carbon black (Super P), 10% carboxymethylcellulose (CMC), and 10% styrene–butadiene rubber (SBR; JSR) by weight. The homogeneous electrode slurry was achieved by vigorous mechanical stirring of the mixture in deionized water. This slurry was then coated onto a cleaned copper foil by tape casting. The coated foil was vacuum-dried at 80 °C for 12 h, followed by roller pressing and precision cutting to obtain 1.5 cm diameter working electrodes with an areal loading of 1.0–1.1 mg cm^–2^. Assembly of CR2032 button cells involved stacking the prepared electrode, formulated electrolyte, a Celgard separator, and a lithium metal electrode in an argon-filled glovebox (MBraun) with ultralow humidity and oxygen levels. The electrolyte contained 1 M LiPF_6_ in EC/EMC (1:2 by volume) with an additional 25 vol % FEC (Novolyte).

### 2.4 Electrochemical Measurements

Charge/discharge profiles and cyclability data were collected from the battery cells using the programmable battery cycler (Lanhe CT3002A). The study employed 2,500 mA h g^–1^ as the rate of 1C. Prior to cycling tests, a 12-h rest period followed cell assembly. The battery underwent discharge and charge cycles between 0.01 and 1.5 V at 0.1C for the initial three cycles, followed by subsequent cycles at 0.5C. Electrochemical measurements, including cyclic voltammetry (CV) and electrochemical impedance spectroscopy (EIS), were conducted using the potentiostat (BioLogic SP-50e). CV scans, ranging from 0.01 to 1.5 V at a scan rate of 0.1 mV s^–1^, were performed, while EIS data were acquired within a frequency range of 1 MHz to 10 mHz, applying an AC voltage magnitude of 10 mV. The resistance of different components was determined by analyzing computer-generated fitted curves obtained from Nyquist plots.

## 3 Results and Discussion

**Figure 1:**
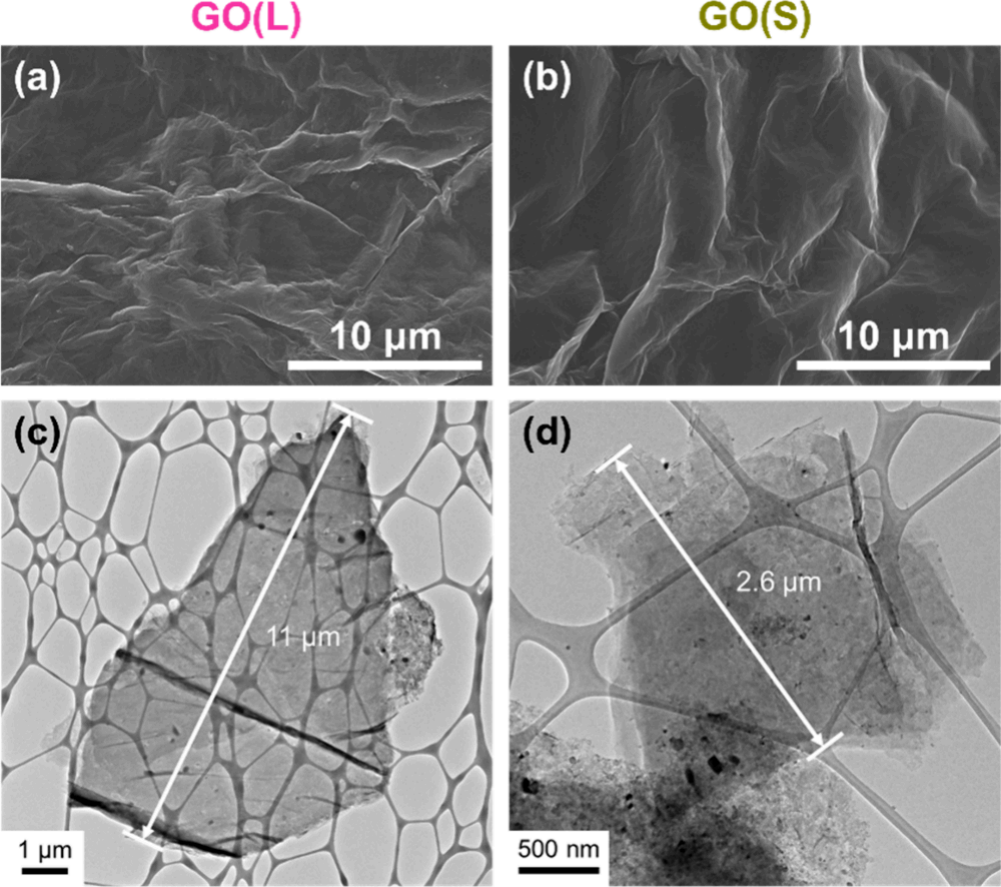
SEM images of (a) large-sized graphene oxide GO(L) and (b) small-sized graphene oxide GO(S). TEM images of (c) GO(L) and (d) GO(S).

This study investigated the effect of tailored GO sizes on fabricating silicon composite anodes for LIBs. Figure 1 comprehensively compares two different GO sizes used in the research. Both GO(L) and GO(S) exhibit a fabric-like structure with a continuous yet naturally wrinkled surface, as observed in Figures 1**a and**1**b**. In Figures 1**c and**1**d**, TEM images show that GO appears very thin and semitransparent. The size of GO(L) is approximately 11 μm, while that of GO(S) is less than 3 μm. DLS particle size measurements provide supplementary size distribution data, as depicted in Figure S1. These results were obtained by considering the diverse projection angles of light onto the suspended GO sheets during the analysis process. Using these two GO materials as surface modifiers, we synthesized reduced graphene oxide-wrapped silicon composite materials (Si@rGO) to elucidate the influence of GO size on the resulting anode properties.

**Figure 2:**
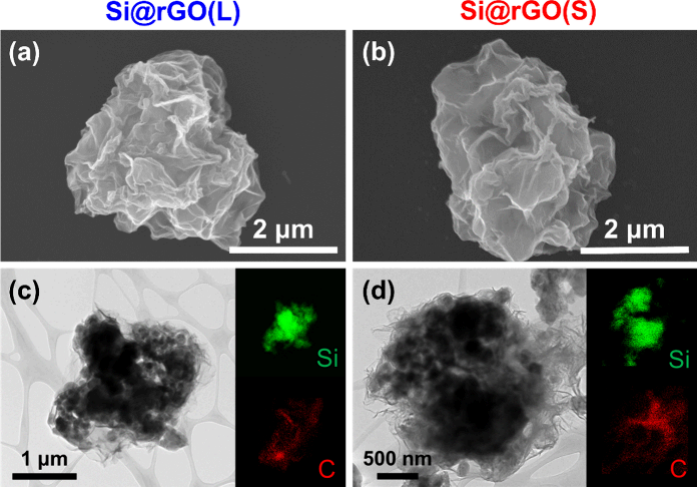
SEM images of (a) Si@rGO(L) and (b) Si@rGO(S) composite materials. TEM images and elemental mapping of (c) Si@rGO(L) and (d) Si@rGO(S) composite materials.

Figure S2 shows that the silicon active material is submicron in size, smaller than the dimensions of both GO sheets, making it suitable for composite fabrication. The synthesis of composite particles consisting of a mixture of Si and GO was achieved by spray drying and thermal reduction. Throughout the spray drying process, droplets containing Si and GO underwent self-assembly to form secondary particles with an average size of about 3–4 μm, as shown in Figures 2**a and**2**b**. The drying process played a critical role in compacting the composite material, resulting in significantly higher tap densities (0.96 g cm^–3^ for Si@rGO(L) and 1.02 g cm^–3^ for Si@rGO(S)) compared to submicron Si (0.37 g cm^–3^). This increased tap density not only contributes to the densification of the composite but also facilitates improved volumetric energy density. TEM images of the composites (Figures 2**c and**2**d**) show a core–shell structure, indicating that the rGO materials serve as the outer shell of the agglomerated silicon particles. TGA data in Figure S3 further illustrate that the carbon content of the Si@rGO composite materials is approximately 7–8 wt %. At about 500 °C, the carbon species begin to decompose, leading to a sharp decrease in mass. From 600 °C – 800 °C, all TGA curves show weight gain, indicating oxidation of silicon in the presence of air.

**Figure 3:**
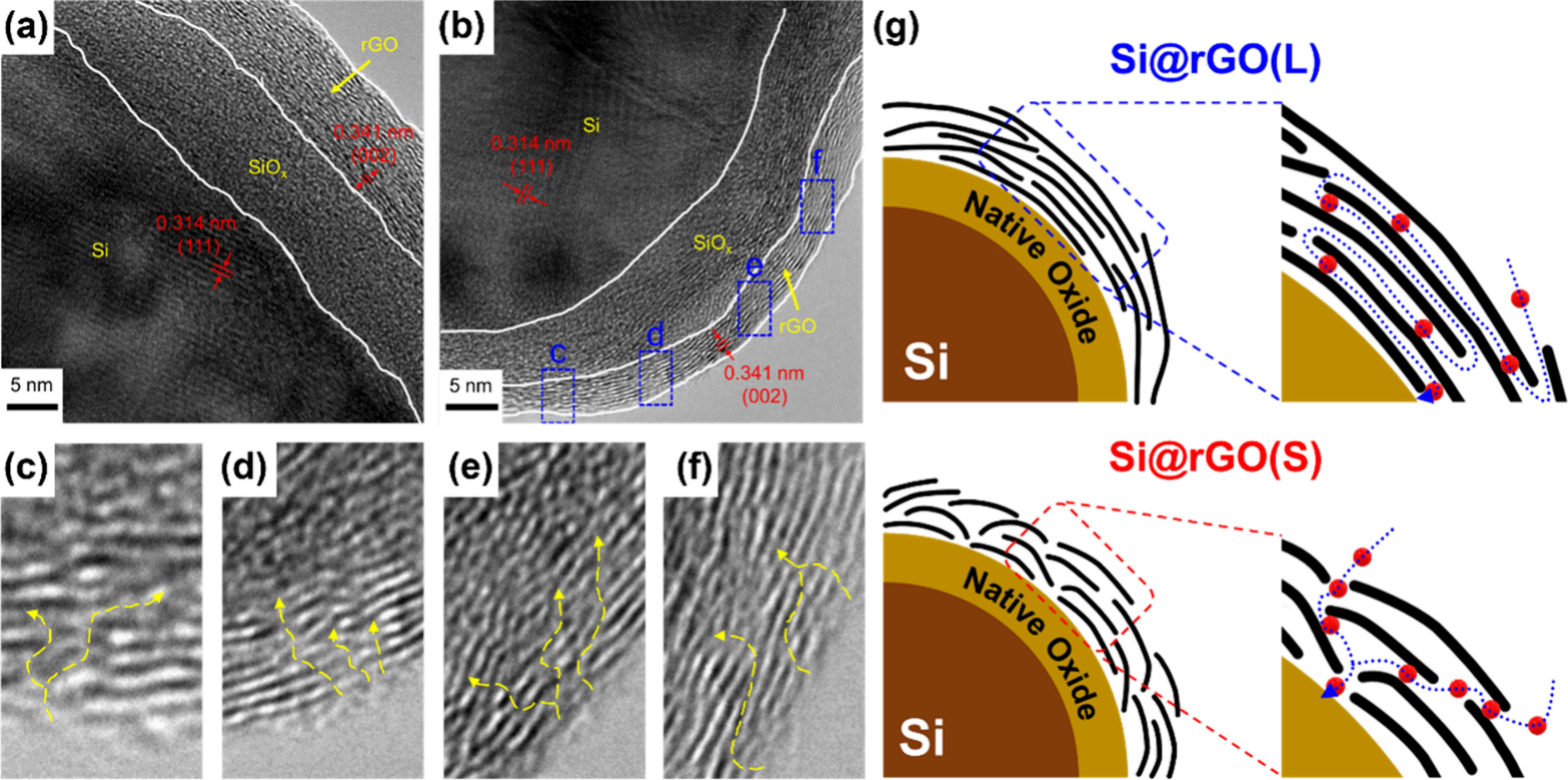
High-resolution TEM images and elemental mapping of (a) Si@rGO(L) and (b) Si@rGO(S) composite materials. (c-f) Magnified TEM images illustrating the accessible Li-ion channels in Si@rGO(S). (g) Schematic drawings of Si@rGO(L) and Si@rGO(S) composite materials depicting distinct Li-ion pathways.

To investigate the composite structure further, high-resolution TEM analysis was performed, as shown in Figures 3**a-**3**f**. The microstructure of the Si@rGO composite materials shows a three-layer architecture with Si as the core, a native oxide layer in the middle, and rGO as the outer layer. The crystallized Si core exhibits (111) visible spacings of 0.314 nm. The native SiO_*x*_ layer is amorphous and approximately 10 nm thick, with some localized crystallization observed in the rGO coating, likely due to potential restacking during the drying and reduction process. Notably, Si@rGO(S) exhibits a higher occurrence of sheet-restacking derived defects on the rGO coating compared to Si@rGO(L), as depicted in Figures 3**c-**3**f**. These defects create multiple Li-ion channels, thereby promoting efficient electrochemical reactions. Conversely, Si@rGO(L) displays pronounced tortuous pathways, as illustrated in Figure S4, which hinder Li-ion diffusion kinetics. Figure 3g illustrates the ion transport paths on Si@rGO(L) and Si@rGO(S) composites, showing that the former requires a longer distance for ion diffusion, while the latter has multiple shorter routes due to the low tortuosity structure facilitated by the smaller size of GO.

**Figure 4:**
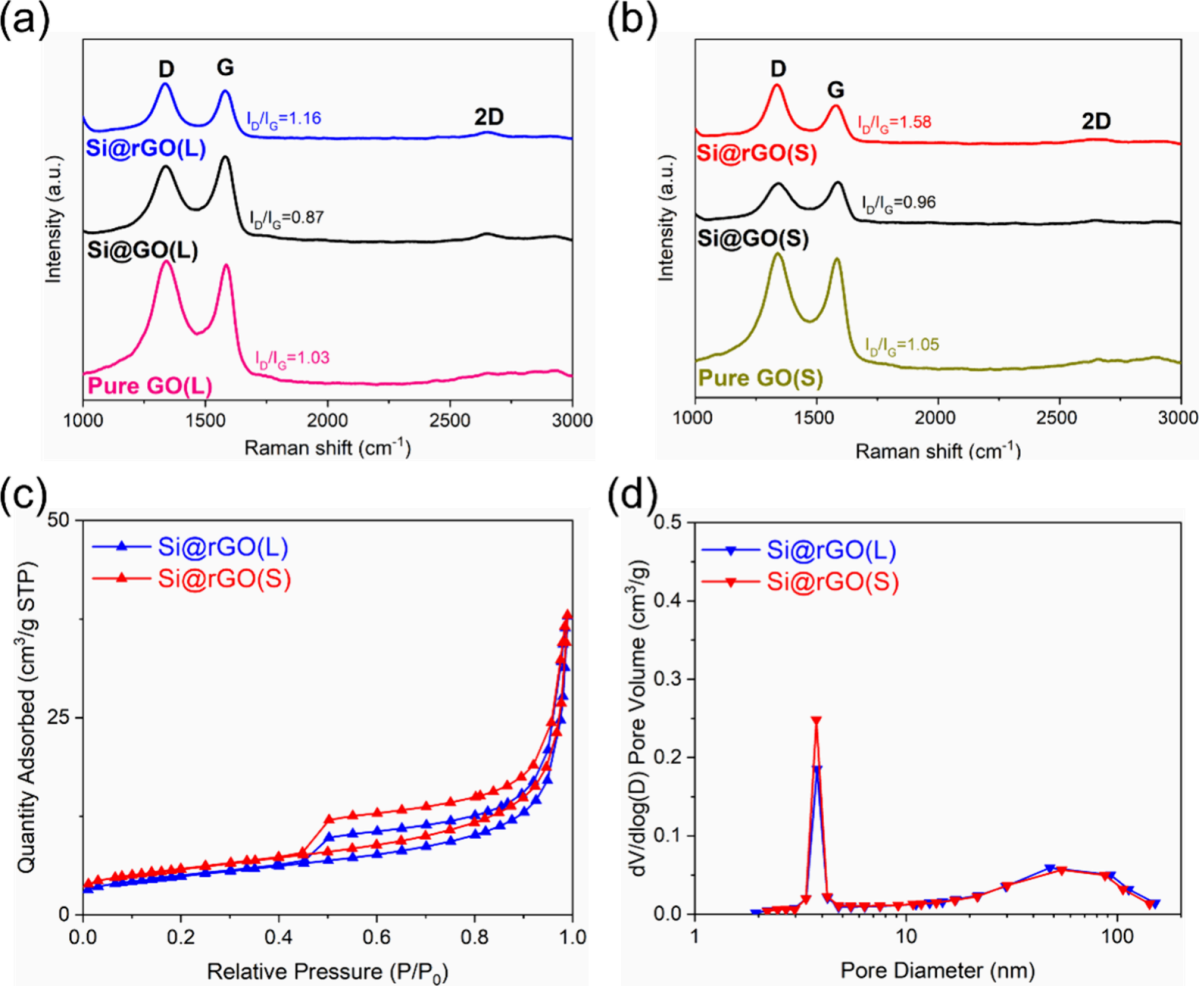
Raman spectra of (a) GO(L), Si@GO(L), Si@rGO(L), and (b) GO(S), Si@GO(S), and Si@rGO(S). (c) Nitrogen adsorption–desorption isotherms and (d) pore size distributions of Si@rGO(L) and Si@rGO(S) composite materials.

The Si@rGO composites were analyzed by XRD, Raman spectroscopy, and BET/BJH methods. In Figure S5, both GO(L) and GO(S) exhibit a small and broad peak at about 12.9°, indicating a large *d*-spacing of 0.69 nm. This significant spacing is due to the oxygen functional groups, especially the basal species covalently bonded to the carbon atoms between the graphene layers.^12^ These interactions occur after overcoming the van der Waals forces of attraction that generally hold the layers together. Upon assembly and reduction in the Si@rGO composites, this peak disappears, probably due to the thin film nature and low content of rGO in the composite. In contrast, the crystalline Si is retained in the Si@rGO composites with Si (111) at 28.7°, Si (220) at 47.6°, and Si (311) at 56.4° based on JCPDS 27–1402. The Raman spectra in Figure S6 also reveal the presence of silicon in the Si@rGO composites, in addition to the characteristic D and G bands of the carbon materials.^6^ In Figures 4**a-**4**b**, the I_D_/I_G_ values serve as an indicator to assess the defect level of rGO in the composites. The pure GO sheets exhibit I_D_/I_G_ values ranging from 1.03 to 1.05. After the spray-drying process, self-assembly of GO layers with silicon micron particles occurs, leading to a reduction in defects, resulting in low I_D_/I_G_ values of 0.87 for Si@GO(L) and 0.96 for Si@GO(S). After thermal treatments, Si@rGO(L) shows a high I_D_/I_G_ value of 1.16, while Si@rGO(S) with a smaller rGO dimension has a much higher I_D_/I_G_ value of 1.58, i.e. more defective, which is consistent with the microstructure analysis in Figure 3. The increased defect level in the Si@rGO composite materials is attributed to the concentration of both basal and edge defects induced by the rigorous reduction process.^6^ In Figure 4c, the BET/BJH analysis of the Si@rGO composites shows type IV adsorption and desorption isotherms,^13^ indicating the mesoporous structure (3–5 nm). Si@rGO(S) shows a higher surface area (20.3 m^2^ g^–1^) compared to Si@rGO(L) (17.3 m^2^ g^–1^) and pure Si (7.01 m^2^ g^–1^), and the former also shows a slightly higher mesopore volume (Figure 4d). The higher tap density observed in Si@rGO composites compared to submicron Si is due to the compact composite structure rather than the influence of surface area. The relatively imperfect restacking of small-sized rGO contributes to the higher surface area and larger pore volume, which facilitates the construction of Li-ion channels and thus could improve the overall electrochemical performance of the composite.

**Figure 5:**
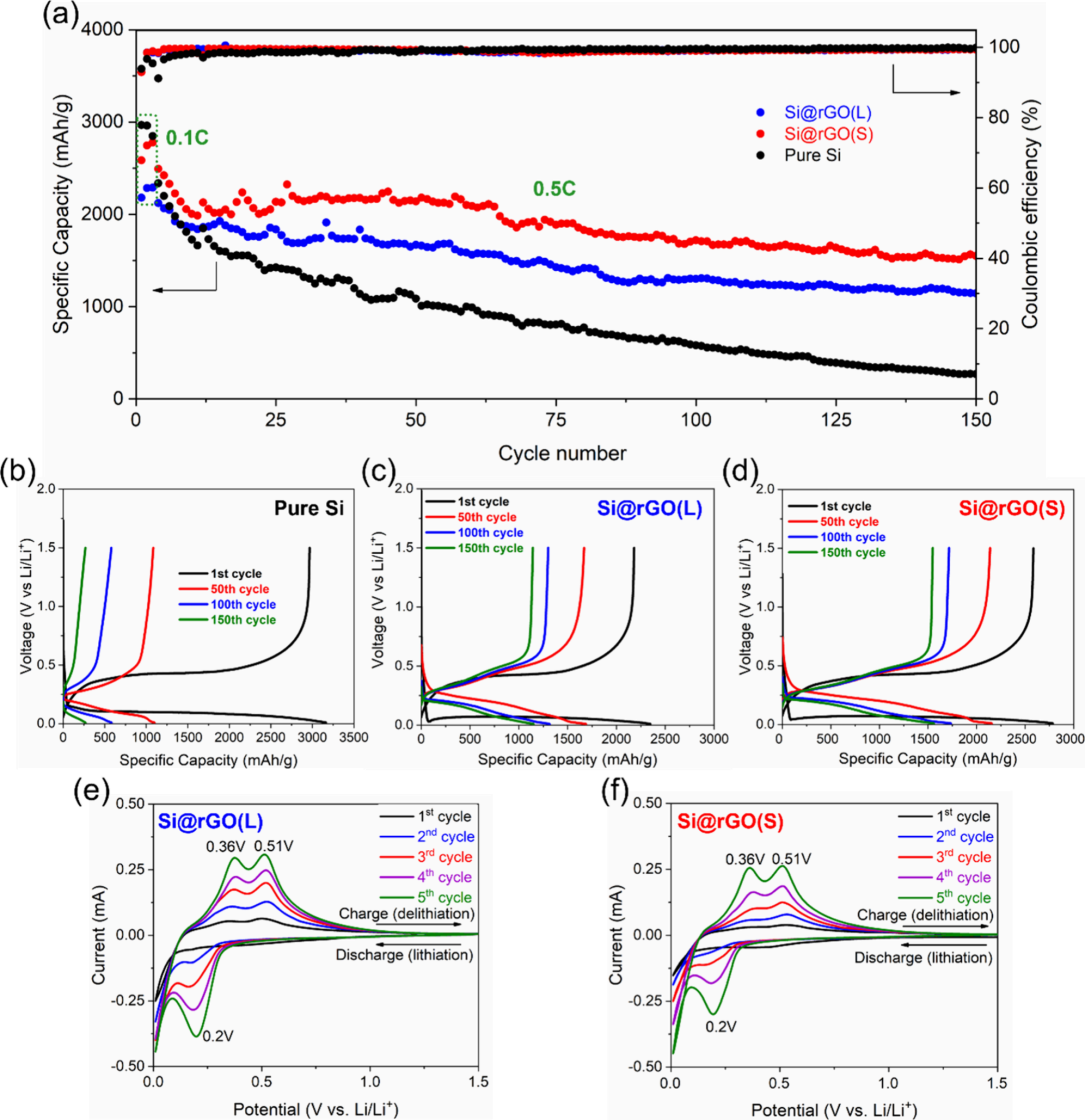
(a) Cycle life data of pure Si, Si@rGO(L), and Si@rGO(S) anodes. Charge/discharge profiles of (b) pure Si, (c) Si@rGO(L), and (d) Si@rGO(S) anode materials. CV plots of (e) Si@rGO(L) and (f) Si@rGO(S) anodes.

In evaluating battery performance, Figure 5a illustrates the cycle life of pure Si, Si@rGO(L), and Si@rGO(S) anodes. When comparing specific capacities, Si@rGO(S) outperforms Si@rGO(L), demonstrating a superior ion channel structure in Si@rGO(S) with a specific capacity of 2,586 mA h g^–1^ compared to 2,181 mA h g^–1^ for Si@rGO(L) at 0.1C. Interestingly, rGO(L) exhibits a slightly higher reversible capacity (211 mA h g^–1^) than rGO(S) (146 mA h g^–1^) when fabricated individually without Si (Figure S7), suggesting that larger graphene basal planes offer more storage sites for Li^+^ ions. However, the majority of the capacity in Si@rGO still originates from active Si due to its significantly higher content and theoretical capacity compared to rGO materials. Both Si@rGO composite materials exhibit great initial Coulombic efficiency approaching 93%, indicating the advantageous nature of the rGO-based composite structure for reversible electrochemical reactions. Additionally, the cycling efficiency of both Si@rGO composite anodes reaches more than 99.5% after the formation cycles, which is significantly higher than the pure Si anode (98.4%). After 150 cycles, the capacity retention rates calculated after the formation cycle for Si@rGO(S), Si@rGO(L), and pure Si are 78%, 62%, and 16%, respectively. This highlights a significant advantage for the Si@rGO(S) composite, which utilizes the interfacial protection of small-sized rGO. This not only provides stable sites for SEI formation, but also offers abundant Li-ion diffusion pathways. The wrinkled rGO also acts as a buffer to accommodate repeated volume changes in the Si material during charging and discharging.^4^Figures 5**b-**5**d** present charge/discharge profiles of Si anodes during cycling. The initial lithiation plateau is low and flat, characteristic of crystalline Si lithiation.^14^ In addition, both Si@rGO composite anodes exhibit an additional overpotential due to the rGO-wrapped Si, which requires an additional driving force for Li^+^ ions to penetrate the rGO coating. After the first cycle, the lithiation curve becomes a sloping shape, indicating the irreversible transition from crystalline Si to amorphous Si.^14^ In contrast to the rapid decay observed for pure Si anodes, the Si@rGO composite anodes exhibit better reversibility, with Si@rGO(S) delivering more capacity after 150 cycles (1,549 mA h g^–1^ for Si@rGO(S) vs 1,144 mA h g^–1^ for Si@rGO(L) at 0.5C).

The CV curves of Si@rGO(L) and Si@rGO(S) anodes are exhibited in Figures 5**e and**5**f**, respectively. A subtle and broad peak centered at ∼0.5 V is observed, representing the initial SEI formation during the first cycle,^15^ which diminishes in subsequent cycles. During the cathodic scan, two prominent peaks at 0.2 and 0.01 V represent the lithiation of amorphous Si (starting from the second cycle) and the subsequent formation of high-order Li_*x*_Si.^16,17^ The anodic scan shows two peaks at 0.36 and 0.51 V, indicating the delithiation of lithiated Si alloys until the formation of amorphous Si.^16,17^ The lithiation and delithiation peaks gradually intensify with each cycle, suggesting continuous activation of the active Si material, consistent with Figure 5a, which shows an increase in capacity during the early cycles.

**Figure 6:**
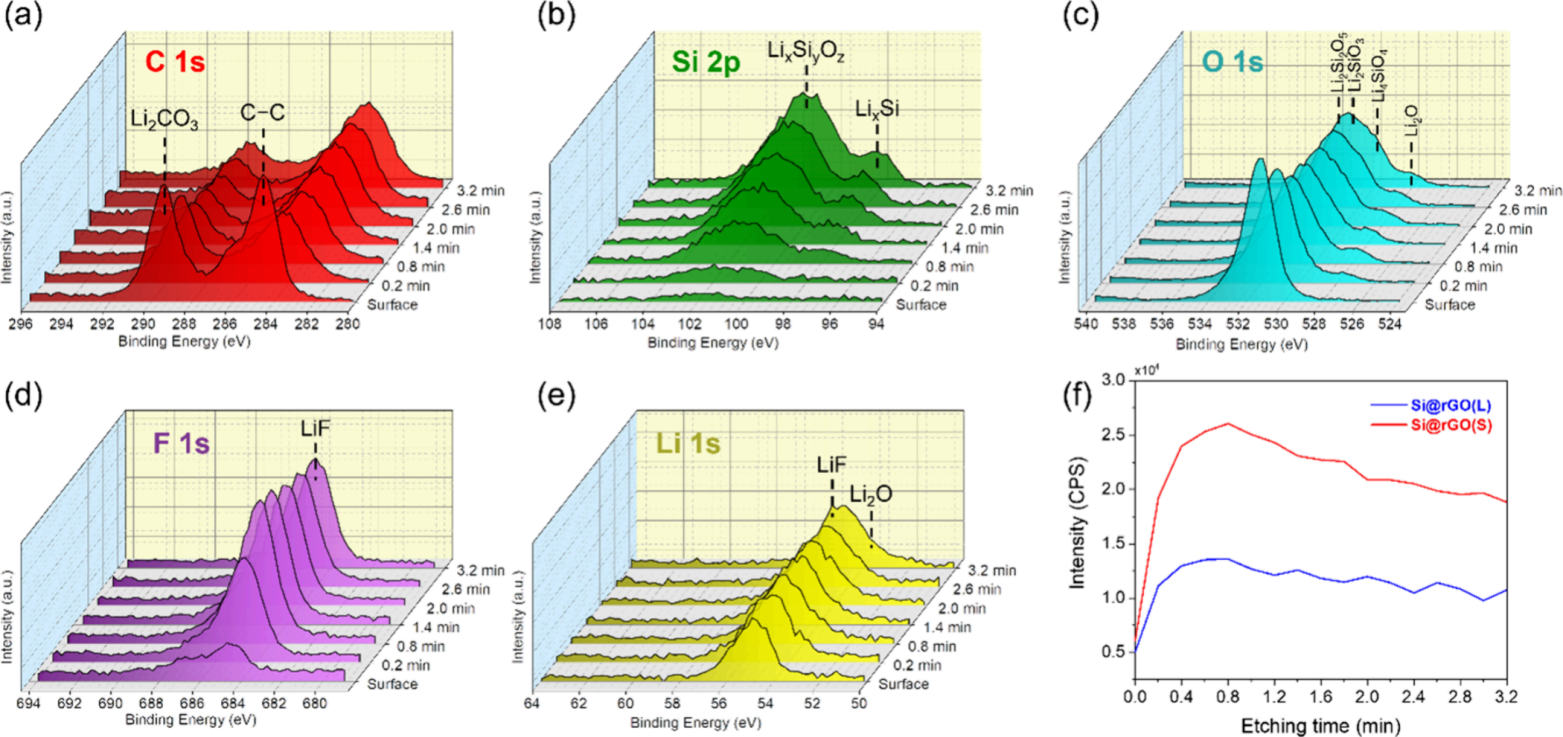
XPS depth profiles of Si@rGO(S) after 50 cycles with different etching times. XPS spectra of (a) C 1s, (b) Si 2p, (c) O 1s, (d) F 1s, (e) Li 1s, and (f) F distribution versus etching time.

In-depth compositional analysis of the Si@rGO composite anodes after cycling was conducted by depth profiling using XPS. Ar^+^ beam etching was applied to reveal the underlying changes by progressively removing material layer by layer. The C 1s spectra (Figures 6**a and**S8a) indicate a reduction in the C–C bond signals at 284.5 eV after the removal of the rGO coating.^18−21^ The residual C signals under the surface are probably attributed to binders and conductive carbon additives. The Li_2_CO_3_ signals at ∼289.3 eV, one of the surface SEI components, also decreases with increasing etch time.^20,21^ The removal of the rGO layer reveals the evolution of Si 2p signals (Figures 6**b and**S8b), starting with the detection of Li_*x*_Si_*y*_O_*z*_ at 101.1 eV from lithiation of the native SiO_*x*_ oxide layer.^22−24^ After 1.4 min of sputtering, the signal of Li_*x*_Si (98.1 eV) appears and intensifies with further etching.^20,22^ The O 1s spectra (Figures 6**c and**S8c) further show the presence of Li_*x*_Si_*y*_O_*z*_ forming high lithiated lithium silicate (Li_4_SiO_4_) near the core and low lithiated lithium silicates (Li_2_Si_2_O_5_ and Li_2_SiO_3_) near the surface.^25−27^ The interfacial inorganic SEI phases Li_2_O (528.1 eV in O 1s) and LiF (684.7 eV in F 1s) (Figures 6**c-**6**e**, **and**S8c-S8e) are detected beneath the top surface,^20−23^ with Si@rGO(S) showing a higher F content compared in Figure 6f, possibly due to abundant sheet-restacking derived defects in the small-sized rGO that promote LiF formation.^28^ The rigid and stable LiF-rich SEI in Si@rGO(S) is proposed to be a crucial factor contributing to its superior cycling stability in LIBs. The distribution of inorganic SEI after cycling based on the XPS depth profile data demonstrates that both LiF and Li_2_O are spread throughout the composite after removal of the topmost organic SEI layer by sputtering,^18,23^ indicating the inward growth of SEI during cycling.^29^

**Figure 7:**
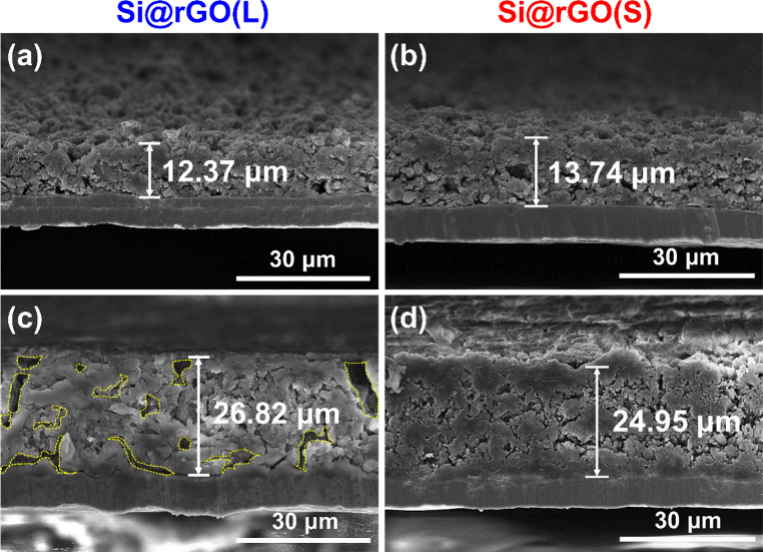
SEM cross-sectional images of (a) Si@rGO(L) and (b) Si@rGO(S) electrodes before cycling, and (c) Si@rGO(L) and (d) Si@rGO(S) electrodes after cycling.

Cross-sectional SEM analysis was performed to evaluate the volume expansion of the electrodes during battery cycling. The initial electrode thickness is approximately 12–13 μm (Figures 7**a and**7**b**). After 100 cycles, Si@rGO(L) exhibits a higher volume change (+117%; Figure 7c) compared to Si@rGO(S) (+82%; Figure 7d), indicating superior swelling control with small-sized rGO. Furthermore, cycled Si@rGO(L) shows numerous voids, highlighted in yellow, while Si@rGO(S) displays a denser structure with a considerably lower defect level, suggesting that small-sized rGO provides better buffering of Si volume changes during lithiation and delithiation, probably due to increased slip planes formed by defects between overlapping rGO layers.

**Figure 8:**
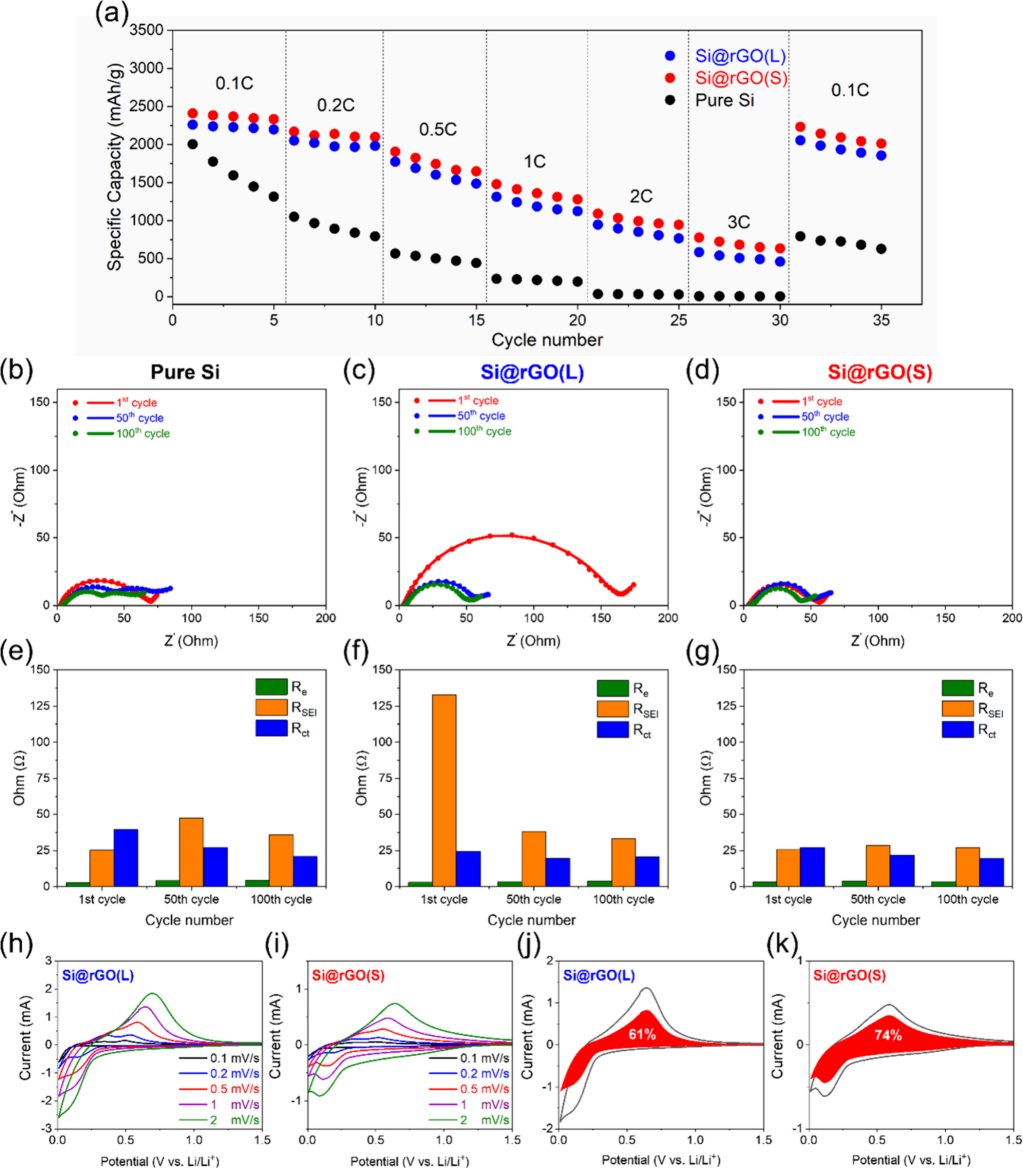
(a) Rate performance of pure Si, Si@rGO(L), and Si@rGO(S) anodes. Nyquist plots of (b) pure Si, (c) Si@rGO(L), and (d) Si@rGO(S) at different cycle numbers. Impedance comparisons of (e) pure Si, (f) Si@rGO(L), and (g) Si@rGO(S) at different cycle numbers. CV scans of (h) Si@rGO(L) and (i) Si@rGO(S) anodes at different scan rates. CV plots showing capacitive controlled contribution at a rate of 1 mV s^–1^ for (j) Si@rGO(L) and (k) Si@rGO(S) anodes.

Figure 8a illustrates the rate capability of pure Si, Si@rGO(L), and Si@rGO(S), showing the superior performance of both Si@rGO composites compared to the unprotected pure Si electrode over a range of current rates up to 3C. To further investigate the factors contributing to the improved cycle life and rate performance of Si@rGO(S), we conducted an EIS analysis, as illustrated in Figures 8**b-**8**g**. The equivalent circuits utilized, depicted in Figure S9, comprised a series of three resistors and constant phase elements (CPE) in parallel, along with a Warburg diffusion element. These elements were employed to delineate various resistances, including electrolyte resistance (R_e_; high-frequency intercept), SEI film resistance (R_SEI_; first semicircle), and charge transfer resistance (R_ct_; mid-frequency second semicircle).^30^ Si@rGO(S) demonstrates consistently low and stable R_SEI_ and R_ct_ over extended cycles (Figures 8**d and**8**g**), contributing to robust cycle life and rate performance due to the LiF-rich SEI and the presence of low tortuous ionic channels.^31,32^ Notably, Si@rGO(L) exhibits significantly elevated R_SEI_ after the first cycle due to the long ionic diffusion path within the highly tortuous large rGO layer (i.e., contributing to the film resistance). After 50 cycles, the repeated swelling of the Si active material mechanically compresses and decompresses the rGO(L) layer, potentially facilitating self-adaptation and SEI reformation, which subsequently reduces its resistance after cycling, although it remains higher than Si@rGO(S). As a result, the small-sized rGO-protected Si composite anode maintains a consistently low impedance over cycles, highlighting the superior design of the reduced graphene oxide-protected Si anode material. CV measurements were conducted at scan rates ranging from 0.1 to 2 mV s^–1^ (Figures 8**h and**8**i**). Analysis using a capacitive charge storage distribution calculation was employed to separate surface capacitive effects and diffusion-controlled processes.^33,34^ At 1 mV s^–1^, Si@rGO(S) exhibits a higher capacitive response than Si@rGO(L), which promotes its fast kinetics (Figures 8**j and**8**k**). Additionally, Figure S10 contrasts the contributions of capacitive and diffusion-controlled charges, revealing a superior capacitive response from the Si@rGO(S) anode across all scan rates, consequently enhancing its rate performance. This comparative analysis, summarized in Table S1, highlights the competitive rate performance of the developed Si@rGO composite anode materials with tailored graphene size, positioning them favorably alongside recently developed graphene-protected Si anodes.

## 4 Conclusions

In summary, this study systematically investigated the effect of tailored GO sizes on the fabrication of silicon composite anodes for LIBs. The Si@rGO composites exhibits a three-layer architecture with silicon as the core, a native oxide layer in the middle, and rGO as the outer layer. The Si@rGO(S) has more defects in the restacked small-sized rGO layers, which provide multiple Li-ion channels to facilitate smooth electrochemical reactions. Battery performance evaluation shows the superiority of Si@rGO(S) over Si@rGO(L) and pure Si anodes in terms of cycle life and Coulombic efficiency. The Si@rGO(S) anode exhibits more stable SEI, higher capacity retention, and better swelling control. The rate capability of Si@rGO(S) surpasses that of Si@rGO(L) and pure Si, demonstrating its improved electrochemical performance. In-depth compositional analysis using XPS depth profiling elucidates the evolution of Si@rGO(S) during cycling, highlighting the formation of a stable LiF-rich SEI, which contributes to superior cycling stability. The tailored GO size in Si@rGO composites significantly influences their structural, compositional, and electrochemical properties. The Si@rGO(S) composite anode emerges as a promising candidate for LIB anodes, demonstrating outstanding performance in cycling stability, rate capability, and impedance characteristics. The results of this study provide valuable insights into the design and optimization of graphene-protected composite materials for the advancement of electrochemical energy storage technologies.
