# Supplementary material for: Tailoring the Size of Reduced Graphene Oxide Sheets to Fabricate Silicon Composite Anodes for Lithium-Ion Batteries
Source: ACS Appl Mater Interfaces. 2024 May 22;16(22):29226–34. doi: 10.1021/acsami.4c03710 (PMC11163395; doi:10.1021/acsami.4c03710)
Supplement: Supplementary file 1 — am4c03710_si_001.pdf [file am4c03710_si_001.pdf]

Supporting Information

Tailoring the Size of Reduced Graphene Oxide Sheets to Fabricate Silicon Composite Anodes for Lithium-ion Batteries

Yun-Zhen Liang[1], Ting-Yu Hsu[2], and Yu-Sheng Su[1,2]*

[1]Industry Academia Innovation School, National Yang Ming Chiao Tung University, 1001 Daxue Road, Hsinchu 300093, Taiwan

[2]International College of Semiconductor Technology, National Yang Ming Chiao Tung University, 1001 Daxue Road, Hsinchu 300093, Taiwan

* Corresponding author at: International College of Semiconductor Technology, National Yang Ming Chiao Tung University, Hsinchu 300093, Taiwan.

E-mail address: yushengsu@nycu.edu.tw

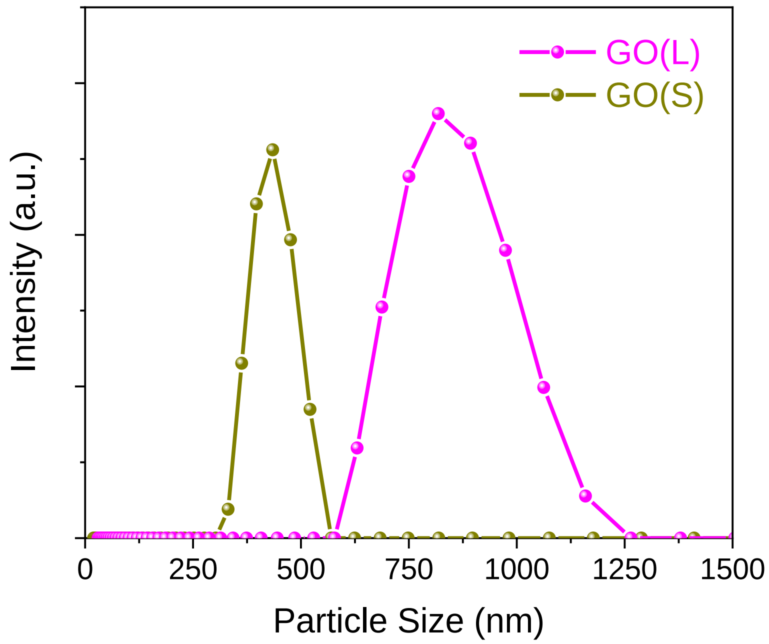

Figure S1. Particle size distributions of GO(L) and GO(S).

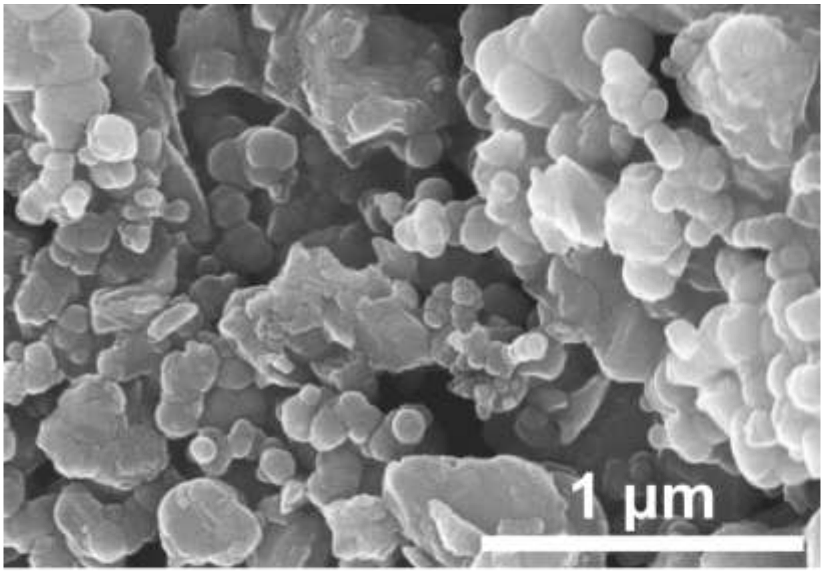

Figure S2. SEM image of pure Si particles.

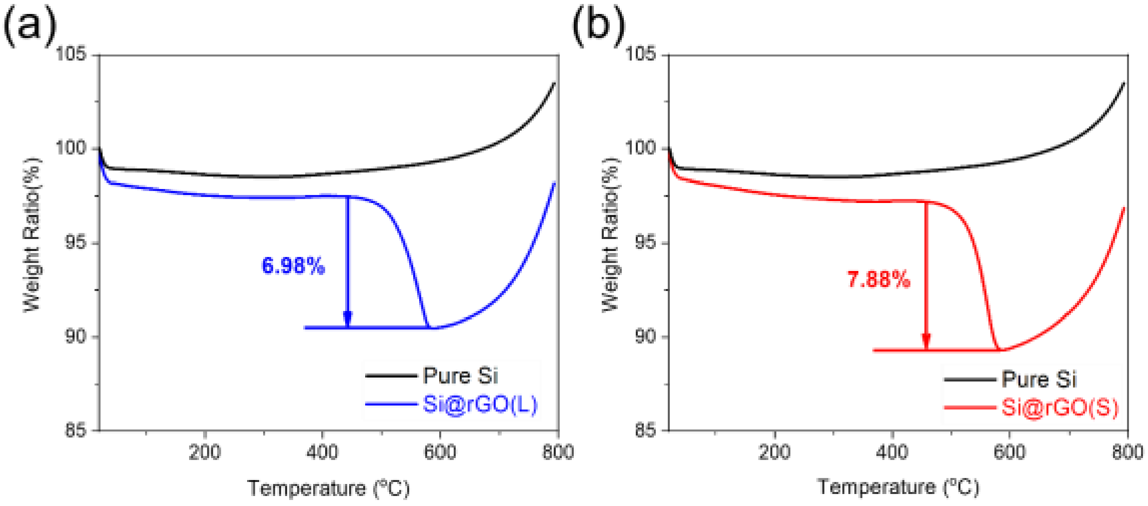

Figure S3. TGA curves of pure Si and (a) Si@rGO(L) and (b) Si@rGO(S) anode materials.

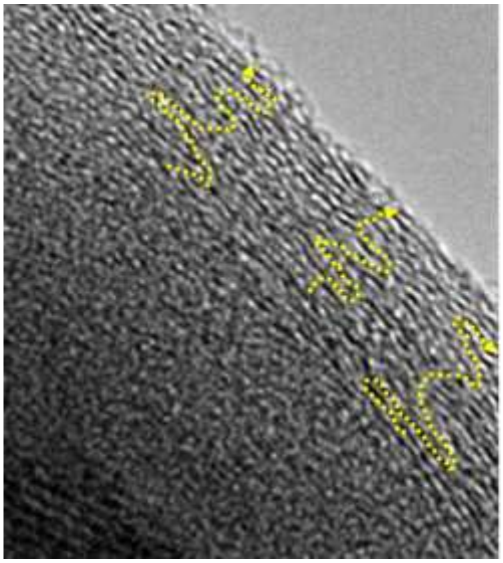

Figure S4. Magnified TEM images illustrating the accessible Li-ion channels in Si@rGO(L).

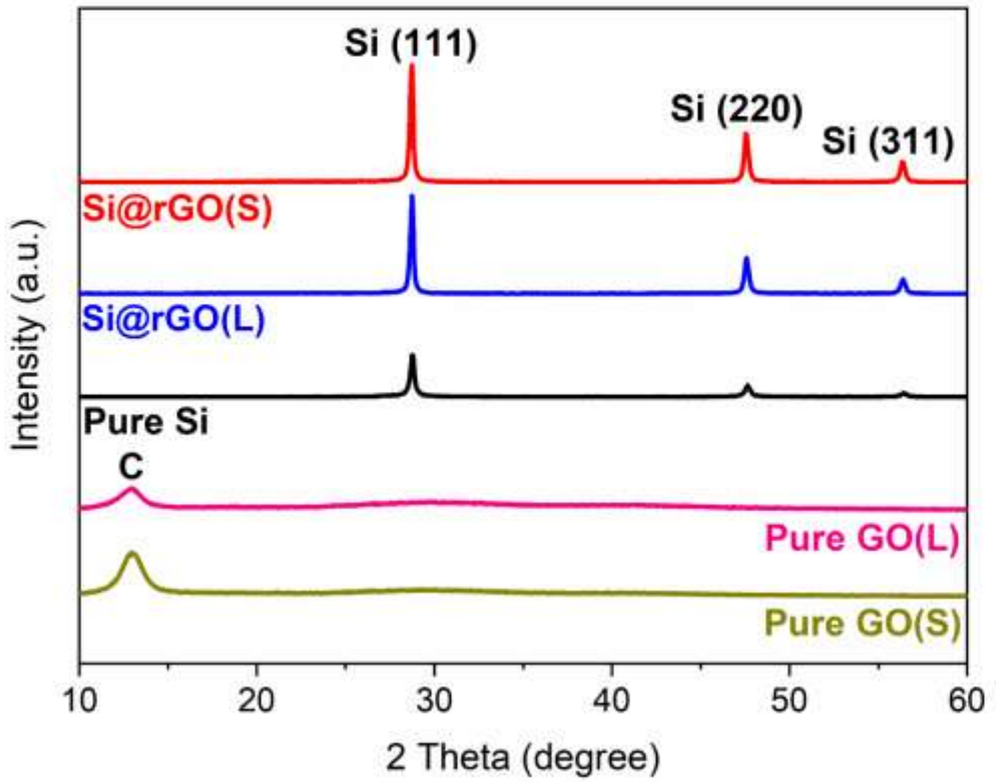

Figure S5. XRD spectra of Si, GO(L), GO(S), Si@rGO(L), and Si@rGO(S) materials.

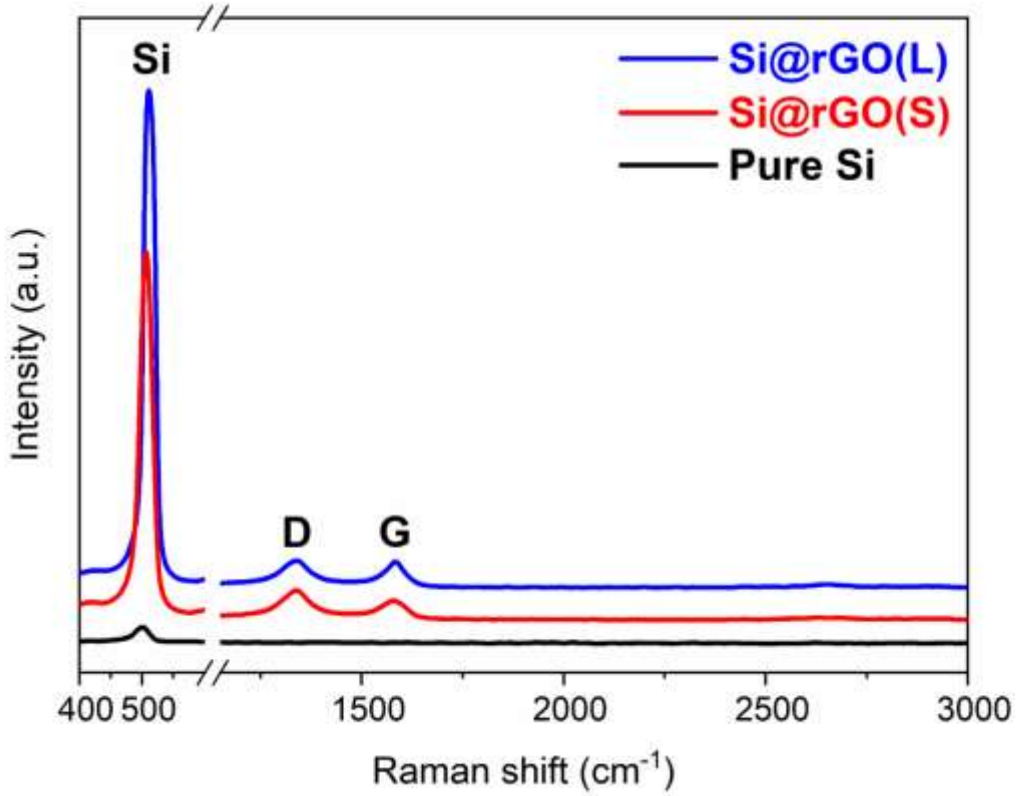

Figure S6. Raman spectra of pure Si, Si@rGO(L), and Si@rGO(S) materials.

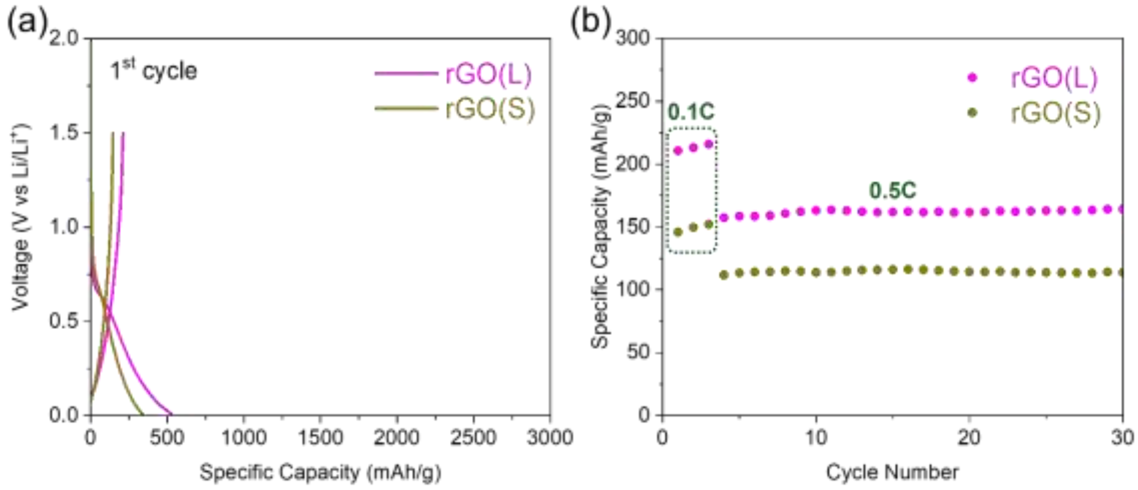

Figure S7. (a) Charge/discharge profiles and (b) cycle life data of rGO(L) and rGO(S) anodes.

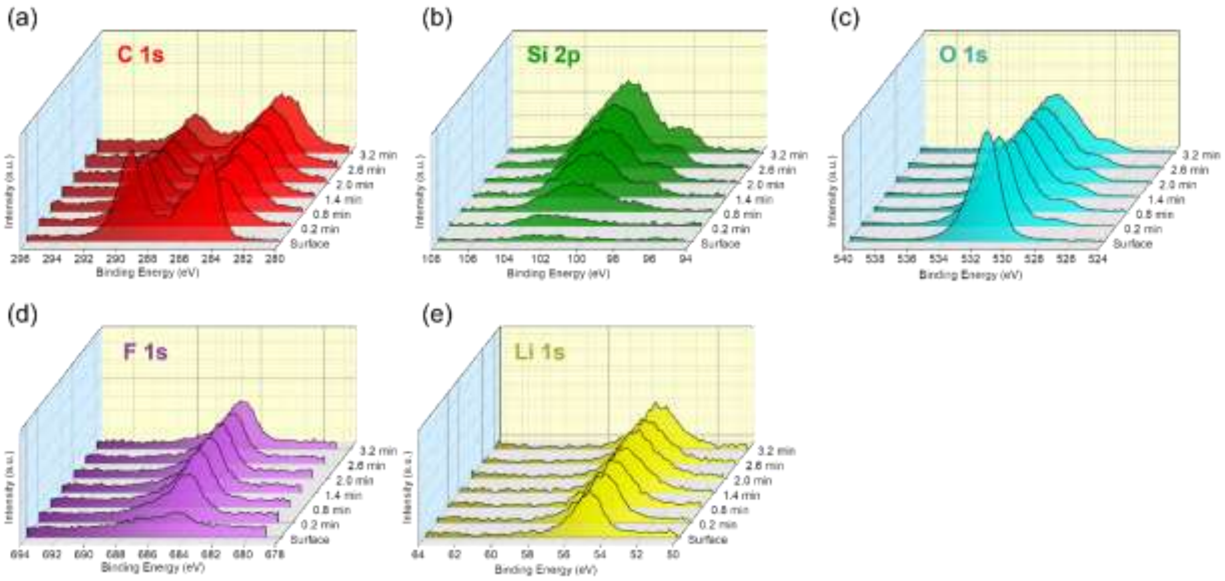

Figure S8. XPS depth profiles of Si@rGO(L) after 50 cycles with different etching times. XPS spectra of (a) C 1s, (b) Si 2p, (c) O 1s, (d) F 1s, and (e) Li 1s.

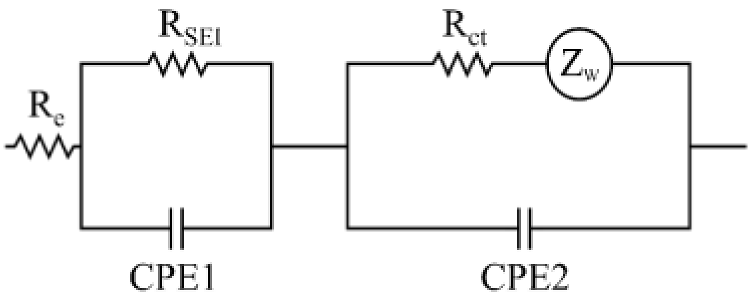

Figure S9. The EIS fitting equivalent circuit model.

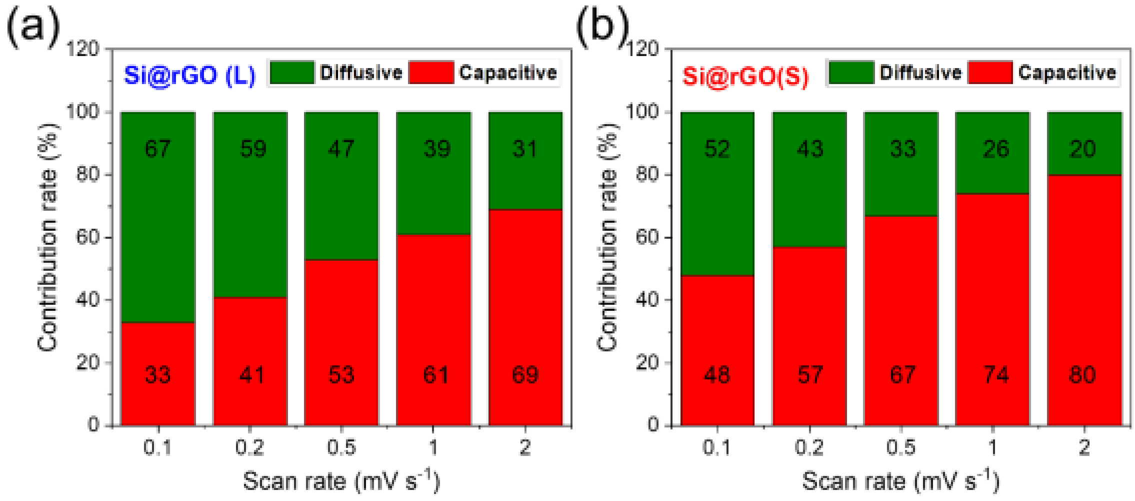

Figure S10. Contribution of diffusive and capacitive charges of cells with (a) Si@rGO(L) and (b) Si@rGO(S) anodes at different scan rates.

Ref.	Reversible Capacity (mA h g^{-1})	Initial Coulombic Efficiency (%)	Capacity Degradation Rate (%/cycle)	Cycle Number	Capacity Available at ≥ 1C	Features
1	932	76.6%	0.078%	300	~700	Binder-free & long cycle life; low-capacity design & low ICE
2	1658	38%	0.12%	100	1027	Graphene/CNT/Si spheres; poor ICE
3	1953	66.9%	0.224%	100	~590	Functionalized ball milling; poor ICE & rate performance
4	2505	86.9%	0.291	100	~1300	Core-shell design; fast capacity degradation
5	~600	75%	0.36%	50	360	Low-capacity design; low ICE & fast capacity degradation
6	2097	90.7%	0.116%	150	706	High-pressure method; poor rate performance
7	821	75.7%	0.04%	100	496	Wet-mix processing; low-capacity design & low ICE
8	345	90%	0.011%	350	NA	3D printing & long cycle life; low-capacity design
9	~2800	~84%	0.165%	100	NA	Camphor-derived graphene
10	2180	79.3%	0.299%	100	NA	Porous Si; low ICE & fast capacity degradation
This work	2586	93%	0.147%	150	1477	Tailored graphene size; good rate performance

Table S1. Comparison of recently developed graphene-protected Si composite anodes for LIBs.

REFERENCES

(1) Shao, F.; Li, H.; Yao, L.; Xu, S.; Li, G.; Li, B.; Zou, C.; Yang, Z.; Su, Y.; Hu, N.; Zhang, Y. Binder-Free, Flexible, and Self-Standing Non-Woven Fabric Anodes Based on Graphene/Si Hybrid Fibers for High-Performance Li-Ion Batteries. *ACS Appl. Mater. Interfaces* **2021**, *13* (23), 27270–27277. https://doi.org/10.1021/acsami.1c04277.

(2) Xu, J.; Yin, Q.; Li, X.; Tan, X.; Liu, Q.; Lu, X.; Cao, B.; Yuan, X.; Li, Y.; Shen, L.; Lu, Y. Spheres of Graphene and Carbon Nanotubes Embedding Silicon as Mechanically Resilient Anodes for Lithium-Ion Batteries. *Nano Lett.* **2022**, *22* (7), 3054–3061. https://doi.org/10.1021/acs.nanolett.2c00341.

(3) Zhang, Y.; Cheng, Y.; Song, J.; Zhang, Y.; Shi, Q.; Wang, J.; Tian, F.; Yuan, S.; Su, Z.; Zhou, C.; Wang, Y.; Yang, S. Functionalization-Assistant Ball Milling towards Si/Graphene Anodes in High Performance Li-Ion Batteries. *Carbon* **2021**, *181*, 300–309. https://doi.org/10.1016/j.carbon.2021.05.024.

(4) Jamaluddin, A.; Umesh, B.; Chen, F.; Chang, J.-K.; Su, C.-Y. Facile Synthesis of Core–Shell Structured Si@graphene Balls as a High-Performance Anode for Lithium-Ion Batteries. *Nanoscale* **2020**, *12* (17), 9616–9627. https://doi.org/10.1039/D0NR01346C.

(5) Feng, Z.; Huang, C.; Fu, A.; Chen, L.; Pei, F.; He, Y.; Fang, X.; Qu, B.; Chen, X.; Ng, A. M. C.; Cui, J. A Three-Dimensional Network of Graphene/Silicon/Graphene Sandwich Sheets as Anode for Li-Ion Battery. *Thin Solid Films* **2020**, *693*, 137702. https://doi.org/10.1016/j.tsf.2019.137702.

(6) Ma, Z.; Wang, L.; Wang, D.; Huang, R.; Wang, C.; Chen, G.; Miao, C.; Peng, Y.; Li, A.; Miao, Y. Crucial Contact Interface of Si@graphene Anodes for High-Performance Li-Ion Batteries. *Applied Surface Science* **2022**, *603*, 154383. https://doi.org/10.1016/j.apsusc.2022.154383.

(7) Li, X.; Li, K.; Yuan, M.; Zhang, J.; Liu, H.; Li, A.; Chen, X.; Song, H. Graphene-Doped Silicon-Carbon Materials with Multi-Interface Structures for Lithium-Ion Battery Anodes. *Journal of Colloid and Interface Science* **2024**, *667*, 470–477. https://doi.org/10.1016/j.jcis.2024.04.113.

(8) Beydaghi, H.; Abouali, S.; Thorat, S. B.; Del Rio Castillo, A. E.; Bellani, S.; Lauciello, S.; Gentiluomo, S.; Pellegrini, V.; Bonaccorso, F. 3D Printed Silicon-Few Layer Graphene Anode for Advanced Li-Ion Batteries. *RSC Adv.* **2021**, *11* (56), 35051–35060. https://doi.org/10.1039/D1RA06643A.

(9) Paravannoor, A.; Deepthi, P.; Kizhakkekilikoodayil Vijayan, B.; Ranjusha, M. K.; Praveen, P. High Capacity and High Rate Capability Si Anodes Encapsulated with Camphor-Derived Graphene for Li Ion Batteries. *Journal of Electroanalytical Chemistry* **2024**, *960*, 118205. https://doi.org/10.1016/j.jelechem.2024.118205.

(10) Huang, Y.; Luo, J.; Peng, J.; Shi, M.; Li, X.; Wang, X.; Chang, B. Porous Silicon–Graphene–Carbon Composite as High Performance Anode Material for Lithium Ion Batteries. *Journal of Energy Storage* **2020**, *27*, 101075. https://doi.org/10.1016/j.est.2019.101075.
